# High-Throughput Line Buffer Microarchitecture for Arbitrary Sized Streaming Image Processing

**DOI:** 10.3390/jimaging5030034

**Published:** 2019-03-06

**Authors:** Runbin Shi, Justin S.J. Wong, Hayden K.-H. So

**Affiliations:** Department of Electrical and Electronic Engineering, The University of Hong Kong, Pok Fu Lam, Hong Kong

**Keywords:** streaming architecture, low-latency, high-throughput, FPGA, D-SWIM, line buffer

## Abstract

Parallel hardware designed for image processing promotes vision-guided intelligent applications. With the advantages of high-throughput and low-latency, streaming architecture on FPGA is especially attractive to real-time image processing. Notably, many real-world applications, such as region of interest (ROI) detection, demand the ability to process images continuously at different sizes and resolutions in hardware without interruptions. FPGA is especially suitable for implementation of such flexible streaming architecture, but most existing solutions require run-time reconfiguration, and hence cannot achieve seamless image size-switching. In this paper, we propose a dynamically-programmable buffer architecture (D-SWIM) based on the Stream-Windowing Interleaved Memory (SWIM) architecture to realize image processing on FPGA for image streams at arbitrary sizes defined at run time. D-SWIM redefines the way that on-chip memory is organized and controlled, and the hardware adapts to arbitrary image size with sub-100 ns delay that ensures minimum interruptions to the image processing at a high frame rate. Compared to the prior SWIM buffer for high-throughput scenarios, D-SWIM achieved dynamic programmability with only a slight overhead on logic resource usage, but saved up to 56% of the BRAM resource. The D-SWIM buffer achieves a max operating frequency of 329.5 MHz and reduction in power consumption by 45.7% comparing with the SWIM scheme. Real-world image processing applications, such as 2D-Convolution and the Harris Corner Detector, have also been used to evaluate D-SWIM’s performance, where a pixel throughput of 4.5 Giga Pixel/s and 4.2 Giga Pixel/s were achieved respectively in each case. Compared to the implementation with prior streaming frameworks, the D-SWIM-based design not only realizes seamless image size-switching, but also improves hardware efficiency up to 30×.

## 1. Introduction

Real-time image processing applications, such as for high-speed image-guided vehicle control [1], requires the underlying image-processing hardware to be both high-throughput and low-latency. Furthermore, for many real-world scenarios, such as in detecting and processing the region of interest (ROI) of arbitrary sizes, the underlying hardware must also be flexible to adapt to the varying input-sized images as needed [2]. With ample high-bandwidth I/O and on-chip programmable logic resources, researchers have demonstrated the benefits of using Field Programmable Gate Arrays (FPGAs) to address the throughput and latency challenges in a wide range of image processing applications. For instance, Wang et al. [3] demonstrated that by using an FPGA to directly process output from a high-speed time-stretch imaging camera, they can successfully classify cell images in real-time with data throughput exceeding 4 Giga Pixels Per Second (GPPS). Similarly, Ma et al. [4] demonstrated an automatic tool for porting general Deep Neural Networks (DNN) to FPGA, which achieves a maximum processing throughput of 710 Giga Operations Per Second (GOPS) and a latency of 31.85 ms for each image frame.

As illustrated by the above examples, one key to achieving high-throughput low-latency image processing on FPGAs is by leveraging carefully pipelined hardware that can operate on the input image as pixel streams without excessive buffering. These hardware architectures are able to commence processing of the image as soon as the necessary pixels are received and continue processing the rest of the arriving image as a pipeline, giving rise to both low-latency and high-throughput operations. Indeed, to facilitate the design of complex streaming image-processing hardware, some FPGA-hardware generators have already been proposed, often relying on the use of domain-specific languages (DSLs) as a bridge between the algorithm designer and the lower-level hardware [5,6,7,8]. In our previous work, SWIM [9], a streaming line buffer generator, was also proposed to address the complexities of rearranging misaligned multi-pixel blocks for ultra high-input throughput applications. It demonstrated that by carefully arranging on-chip memory resources to align with the input image size, a fully pipelined image processing system on FPGA could be realized that operates close to the FPGA maximum clock frequency.

However, while these hardware generation frameworks can efficiently produce designs for a particular target application, they must be pre-configured to a fixed input image size before the FPGA synthesis. The FPGA has to be reconfigured when the input image size changes, limiting their use in real-time applications that operate on input images with varying sizes.

Building on top of the work of SWIM, we present in this paper an improved high-throughput hardware architecture that can adapt to the size of the input image dynamically during runtime without hardware reconfiguration. The improved scheme, called Dynamic-SWIM (D-SWIM), utilizes an improved on-chip memory organization that can adapt to changing the image size dynamically. Different to SWIM, the D-SWIM framework generates light-weighted control instructions for different image sizes. The hardware architecture can be rapidly programmed in sub-100 nanoseconds instead of seconds to half a minute of FPGA reconfiguration, making it suitable to process images of different sizes seamlessly. Such dynamic programmability with D-SWIM is achieved with only a slight overhead on logic resource usage. Furthermore, D-SWIM lowers overall power consumption by 45.7% due to reduced BRAM usage. This paper also provides a D-SWIM based hardware design method with two real-world applications as a case study.

The rest of the paper is organized as follows: Section 2 presents the basis of streaming architecture and the motivative scenarios of high-throughput and arbitrary sized image processing. Section 3 describes the D-SWIM framework, including the hardware structure and instruction compilation for any image size. Section 4.2 gives the logic implementation of the fully pipelined hardware. We deeply evaluated the D-SWIM with practical image applications. Section 5 shows the experiments and the results compared to SWIM and other streaming architectures. Section 6 is the conclusion.

## 2. Background

### 2.1. Streaming Architecture for Image Processing on FPGA

Similarly to the traditional computer system, memory hierarchy exists in FPGA-centric systems. On-chip memory inside the FPGA has low access latency, but relatively small capacity. In contrast, off-chip memory (DRAM) has a larger capacity, but longer latency and lower bandwidth. Furthermore, DRAM access consumes significantly more energy than on-chip memory. Therefore, in the field of FPGA architecture for image processing, it is a hot topic to trade off the on-chip buffer cost and system performance. For streaming architecture, it is widely adopted that the FPGA receives the pixels line-by-line as they are captured by the image sensor. The on-chip buffer is employed to store multiple lines for the 2D pixel access in the computation. Note that the buffer is optimized to the minimum size, and only the stores the pixels if they will be reused in subsequent computations.

**Figure 1:**
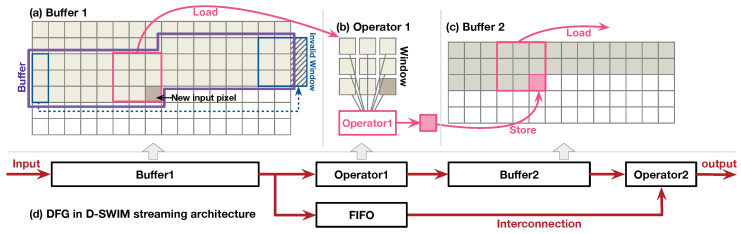
A streaming architecture example for image processing with a 2D pattern. The architecture has three components: buffer, operator, and interconnections.

Previous works presented general methods for designing a streaming architecture for image processing with a 2D access pattern [5,7,10]. Figure 1 shows an example. There are three components within this streaming architecture: *Buffer* (BUF), *operator*, and *interconnections*. The BUF stores multiple image lines that arrive sequentially in a line-by-line manner from the input stream, and the *operators* can simultaneously access pixels across multiple lines within a local area defined by a 2D window or stencil pattern. For instance, in Figure 1, the operator 1 (OP1) performs 2D filtering with a 3×3 sliding window, and the step size of sliding window is one pixel in both vertical and horizontal directions. Assuming the FPGA receives one new pixel from the input stream per cycle to sequentially fill the input Buffer 1 (BUF1). Concurrently, BUF1 outputs 3×3 pixels in a window that is needed by OP1 to produce one resultant pixel. In each clock cycle, the window advances to the right by one step, and the output pixel is stored in BUF2. Note that while the window is moving to the boundary of each line, the output window of BUF1 concatenates the pixel columns from both the end and start in the buffer, as Figure 1a shows. The windows across the boundary are invalid, and the corresponding resultant pixels are dropped, such that the line width of BUF2 is less than the width of BUF1.

As illustrated, BUF1 dynamically maintains three image lines that will be reused. Note that the on-chip BUF can be optimized to a minimum size where the new pixels are consumed as soon as they become available in the BUF and the old pixels are discarded. The BUF design for the other 2-D stencil patterns follows a similar principle. *Operator* is composed of arithmetic units (adder, multiplier, etc.) tailored for the image application. The results from the operators can be either output as the final result, or stored in another BUF which provides the input of the subsequent operator. The *interconnections* are the dedicated data paths that follows the data flow graph (DFG) of the application. For instance, in Figure 1d, Operator 2 (OP2) uses both the pixels in the initial BUF1 and the output of OP1 stored in BUF2 for further processing. In addition to the direct wires, *first-in, first-out (FIFO)* was inserted on the data flow path to guarantee all pixels of the required pattern arrived at the operator in the correct clock cycle.

### 2.2. Demand on Arbitrary Sized Image Processing

**Figure 2:**
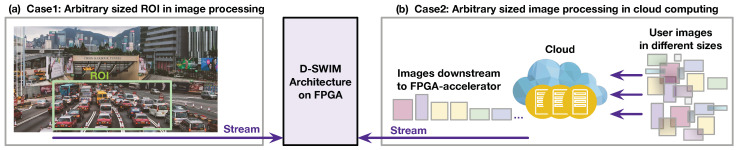
Motivation for arbitrary sized image processing: (**a**) user-defined Region of Interest (ROI) processing; (**b**) arbitrary sized image processing in cloud computing.

In many real-world image processing scenarios, the size of the image is unpredictable before the system run-time. To demonstrate, Figure 2 presents two example cases.

The first case is the Region of Interest (ROI) processing. As Figure 2a shows, the ROI is selected from the entire view of the image for analysis. This mechanism exists in most image applications that effectively reduce the computation workload. However, the ROI is defined by the end-user—hence, the size of ROI is unpredictable during the hardware design time. Furthermore, multiple ROIs may exist on the same view, such that the hardware is required to accommodate images of different sizes in processing one frame.

The second case presents how the arbitrary size image processing is also demanded in cloud computing. As Figure 2b shows, the users at the edge side upload images to the cloud for the computation-intensive applications (such as inference of deep learning, etc.). The cloud server sends the workload to the FPGA accelerator to reduce CPU processing time.

In both cases, the streaming architecture on FPGA is required to process arbitrary sized images. Furthermore, the working mode of hardware should be quickly switched for seamlessly processing the images. The conventional FPGA reconfiguration costs seconds to half a minute, which greatly reduces the system efficiency. Thus, we investigate a streaming architecture that can be rapidly programmed to process images in an arbitrary size.

### 2.3. Demand on Ultra-Fast Stream Processing

In previous works, the FPGA streaming architectures accept pixel streams with a throughput of one or two pixels per clock cycle (pix/cycle) [5,7]. Due to the fast-growing bandwidth of peripherals, demand comes that FPGA should process multi-pixel blocks instead of independent pixels in each cycle. For instance,

- **Near-storage processing:** High-bandwidth memory (HBM) stacks multiple DRAM dies to achieve a bandwidth of 250 GByte/s [11]. Assuming the operating frequency of FPGA is 250 MHz, the max data rate of a FPGA input stream is 1000 Byte/cycle. For images with 1 byte per pixel, this translates into a pixel throughput of 1000 pix/cycle.
- **Near-sensor processing:** The high-resolution image sensor represents a high pixel throughput. For instance, the up-to-date CMOS sensor Sony IMX253 is capable of capturing 68 frames per second, with a resolution of 4096×3000 [12]. Thus, the minimum processing throughput on FPGA is 4 pix/cycle (⌈4096×3000×68/250MHz⌉).

### 2.4. BRAM-Misalignment Challenge and SWIM Framework

**Figure 3:**
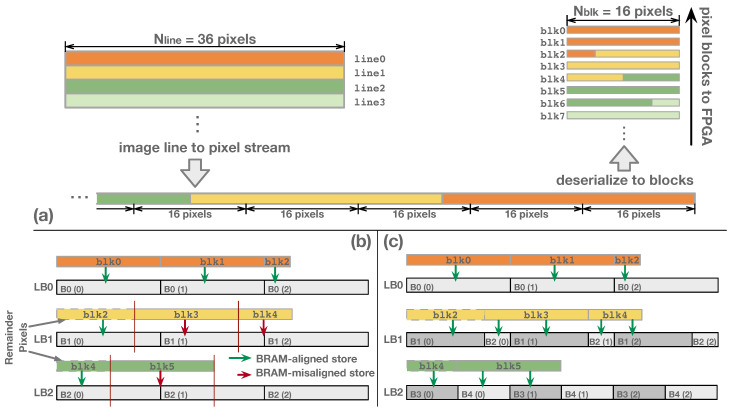
(**a**) Shows that the image lines are sequenced into a stream and then clipped to multi-pixel blocks; FPGA accepts one block in each cycle. (**b**) Shows the general pixel buffer in which the BRAM-misalignment issue occurs. (**c**) Shows the SWIM buffer avoids the BRAM-misalignment using specific BRAM partition.

An increase in processing throughput demands a more complex buffer that relies on the parallel pixel access using multi-pixel blocks. This, however, introduces potential memory alignment issues when utilizing BRAMs in the buffer design. An example in Figure 3a illustrates this problem, where the original image lines are sequenced into a high-throughput 1D pixel stream, and then clipped to pixel blocks by the serial-to-parallel hardware (deserializer) inside the FPGA. The image-processing logic accepts one pixel block in each cycle. Complication due to memory block misalignment arises when the pixel number of one image line (denoted as $N_{line}$) is not an integer multiple of the pixel number in an input block ($N_{blk}$). In this case, some of the blocks ended up encapsulating pixels from two image lines. As an example, in Figure 3a, we have $N_{line}=36$ and $N_{blk}=16$. Thus, blk2, blk4, blk6, at the end of line0, line1, line2, contains 12, 8, and 4 pixels, respectively, that belong to the start of the next line. These pixels are labeled as *remainder pixels* in the example. As Figure 3b shows, the general pixel buffer is composed of multiple line buffers (LBs) and each LB stores an entire image line. The LB is implemented with one BRAM with a size of $N_{blk}$ to fulfill the parallel pixel access. We annotated the BRAM index (*n*) and the address (addr) in the diagram as Bn(addr) to present the storage pattern. Note that the remainder of the pixels within the last block of each line will be stored in the following LB. Therefore, the storage of subsequent blocks may inherit an alignment offset relative to the BRAM boundary. For example, in Figure 3b, the last block of line0(blk2) contains 12 remainder pixels that are written to LB1. To store the blk3 continuously in LB, two addresses of LB1 (B1(0),B1(1)) are accessed. However, this behavior overwrites the pixels of blk2 stored in B1(0).

To address the misalignment issue, Wong et al. [9] proposed SWIM, a BRAM partition method for the pixel buffer design. With the same case, Figure 3c shows the SWIM buffer architecture and the pixel storage pattern. Each LB is composed of two BRAMs, and the width of the first BRAM is equal to the number of remainder pixels. For example, LB0 is composed of only one BRAM because there is no remainder pixel at the end of the previous line; LB1 is partitioned into BRAM1 and BRAM2 with widths of 12 and 4, respectively. Thus, the 12 remainder pixels in blk2 are stored at B1(0), and blk3 are stored separately at B2(0) and B1(1). With this method, SWIM guarantees that the block storage is aligned to the BRAM boundary. Although the SWIM framework generates BRAM partition configurations that avoid the BRAM-misaligned access, the hardware configuration needs to be re-generated through FPGA synthesis flow for a different image width ($N_{line}$). Even if the FPGA configuration files for different $N_{line}$ can be pre-generated before the run-time, the long disruption caused by FPGA reconfiguration for differently sized images significantly decreases the throughput.

## 3. Method

This section describes the Dynamic-SWIM (D-SWIM), a flexible buffer architecture that can be rapidly reconfigured to accommodate arbitrary sized images via instruction updates. First, an overview of the D-SWIM framework (hardware-, software-tools, and design parameters) is given; then, the hardware of D-SWIM is described. Subsequently, the control method of D-SWIM and the custom instruction-set are described, followed by the system working-flow illustration.

### 3.1. Framework Overview

**Figure 4:**
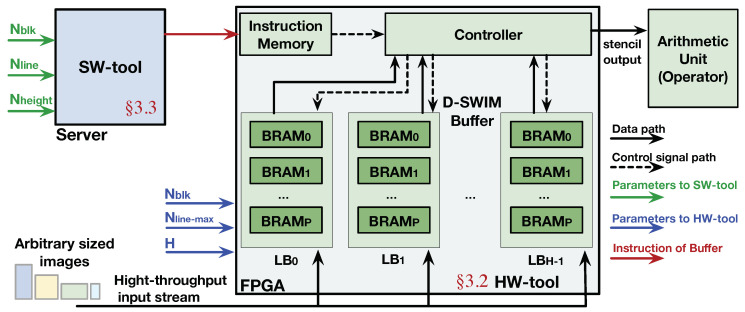
Overview of D-SWIM framework.

As shown in Figure 4, the D-SWIM framework is composed of two parts: the *software compilation tool*, (SW tool), and *hardware generation tool* (HW tool). The HW tool generates hardware components following the D-SWIM method. Note that D-SWIM mainly optimizes the buffer hardware which is a general component of the streaming architecture, where specific arithmetic units (operators) are generated by FPGA synthesis tools without further optimization. According to the principle of stream processing in Figure 1, the Buffer of D-SWIM is composed of multiple line buffers (LBs), and the number of LB ($N_{LB}$) is equal to the height of a 2D stencil (denoted as *H*). Details on the construction of LBs will be elaborated in Section 3.2. Inside the buffer hardware, the Controller module provides the control signals to the underlying BRAMs to realize certain buffer behavior. Note that we employed an InstructionMemory to provide the control words which can be pre-generated and loaded into the memory during run-time. By doing so, the D-SWIM hardware can accommodate the arbitrary image size by quickly switching the control words in the InstructionMemory in a few cycles instead of performing FPGA reconfiguration. The SW tool generates the specific instruction words for the Controller based on the pixel block size and image size, that will be described in Section 3.3.

The design parameters used in D-SWIM are listed in Table 1. $N_{blk}$ is the number of pixels in one block; $N_{line}$ is the number of pixels in one image line (image width), and $N_{height}$ is the image height; $N_{line-max}$ is the largest possible value of $N_{line}$ that decides the volume of the Buffer; and *H* is the height of the 2D stencil pattern that determines the number of image lines stored in the buffer (number of LB). Table 1 also highlighted the use scope of each parameter. Note that the HW tool only invokes $N_{blk}$, $N_{line-max}$, and *H*, which are independent to the image size.

### 3.2. Buffer Architecture in D-SWIM

#### 3.2.1. BRAM Organization of Line Buffer

Similarly to SWIM, the D-SWIM buffer is composed of BRAM that saves the FPGA hardware resource and avoids the complex routing requirement. This section describes the BRAM-centric technique for the D-SWIM buffer construction.

**Figure 5:**
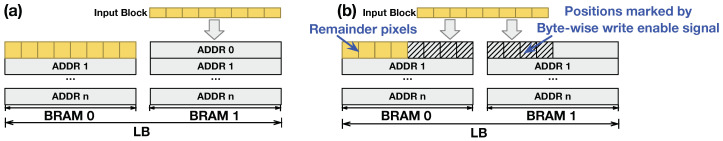
(**a**) shows LB write behavior with conventional BRAM usage. (**b**) shows LB write behavior with the byte-wise write enable signal using BRAM primitive instantiation.

**BRAM Configuration:** D-SWIM directly employs the BRAM primitive for the buffer. The port width of BRAM can be configured. To accommodate the parallel pixels access of the input block and fully utilize the BRAM bandwidth, we configured all the BRAMs to a port width of 64 bits in the simple dual-port (SDP) mode. Note that the real maximum port width is 72 bits, whereas only 64 bits are under the control of the byte-wise write-enabling signal. The conventional usage of BRAM considers all bits in one address as an element in memory access. As Figure 5a shows, the store of the input block should align to the BRAM boundary—otherwise, the misaligned store will overwrite the other bits in the same address. To avoid interference between two consecutive blocks which is misaligned with the BRAM, we used the BRAM primitive instantiation within Xilinx Vivado that provides a *byte-wise write-enable signal* [13]. For instance, in Figure 5b, the input block (8 pixels, 8 bits/pixel) is misaligned to the BRAM boundary because the 4 pixels in the head of BRAM0 are stored along with the previous block. With the byte-wise write enable, the specific control bits in BRAM0 and BRAM1 are set to indicate the store positions of the incoming 8 pixels and the 4 remainder pixels ahead will not be overwritten. In summary, with the BRAM primitive instantiation, the controlling of the pixel-block store becomes more fine-grained. Furthermore, the write enable signal can be changed in the FPGA run-time such that the LB accommodates arbitrary writing offset introduced by the remainder pixels.

**Number of BRAM in LB:** The number of BRAM invoked by one LB (denoted as $N_{bram}$) is determined by the system parameters in Table 1. D-SWIM targets minimizing the BRAM consumption under the constraints in Equation (1). Firstly, the capacity of the LB should be larger than $N_{line-max}$. Secondly, the overall port width of the LB should be large enough to tackle the pixel block and ensure that only one address in each BRAM will be accessed in one cycle, such as the minimum $N_{bram}$ is 2 in the Figure 5b case. Otherwise, two addresses of BRAM0 will be accessed in the same cycle that violates the port limitation of BRAM in the SDP mode.

(1) $$\begin{array}{cc} \underset{}{\mathrm{minimize}} & N_{bram}\\ \mathrm{subject}\;\mathrm{to} & N_{bram}\times D_{bram}\times W_{bram}\geq N_{line-max}\times 8\left(bits/pix\right)\\ & N_{bram}\times W_{bram}\geq \left(N_{remain-max}\%\left(W_{bram}/8\right)+N_{blk}\right)\times 8\\ & N_{bram}\in Z_{>0}. \end{array}$$

In Equation (1), $D_{bram}$ and $W_{bram}$ is the depth and width of a BRAM that is equal to 512 and 64, respectively, in Xilinx FPGA. $N_{remain-max}$ is the largest possible number of the remainder pixel which is equal to $N_{blk}-1$. % is the modulo operation. Therefore, we obtained the value of $N_{bram}$ as Equation (2).

(2) $$N_{bram}=\max\left(N_{line-max}/4096,\left\lceil \left(\left(N_{blk}-1\right)\%8+N_{blk}\right)/8\right\rceil \right)$$

#### 3.2.2. Line-Rolling Behavior of Line Buffers

**Figure 6:**
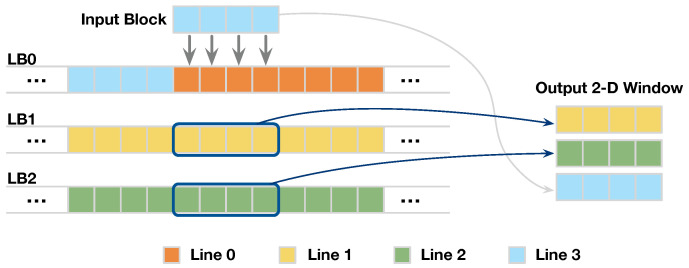
Example of buffer load and store with the line-rolling behavior.

The buffer in D-SWIM stores and loads the successive image lines with the line-rolling mechanism. To demonstrate the line-rolling clearly, we show an example with $H=3,N_{blk}=4$ in Figure 6. At the beginning, the LB0-LB2 are stored in the Line0-Line2, respectively. When the buffer receives the incoming Line3, the input block is stored in LB0 and replaces the old pixels of Line0 which is no longer needed. With this *line-rolling* mechanism, the successive image lines are stored in all LBs in a cyclic manner.

Meanwhile, the buffer outputs a 2D window including pixels from *H* image lines, and the blocks in the window are spatially aligned to the input block. As Figure 6 shows, the first two blocks of the output window are loaded from the LBs, while the last one is directly sourced from the input block. Because the output blocks are aligned in the vertical direction, the 2D windows are continuous in the horizontal direction. Thus, the output 2D windows cover all the pixels required by an arbitrary 2D pattern with a height of *H*.

### 3.3. Line Buffer Access Pattern and Control Instruction

#### 3.3.1. Access Pattern of Line Buffer

**Figure 7:**
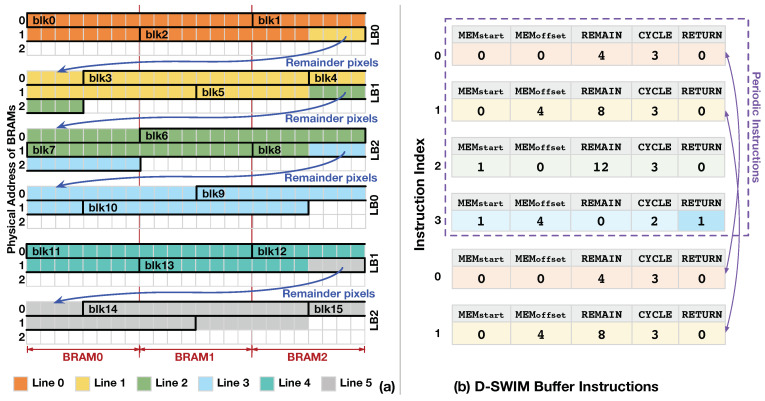
(**a**) shows an example of the block storage pattern with parameters $N_{line}=44$, $N_{blk}=16$, and H=3. (**b**) shows the buffer instruction list for achieving the access pattern in (a).

Since the image lines are vertically aligned in the LBs, the load addresses are synchronized to the store address of the LB which accepts the input block. We use an example in Figure 7 to demonstrate the pixel access pattern in the underlying BRAMs. The parameters, $N_{line}$, $N_{blk}$ and *H* are set to 44,16,and3, respectively in the example. With the D-SWIM method, we set 3 LBs (H=3) in the streaming architecture, and each LB is composed of 3 BRAMs ($N_{bram}=3$). The store position of input blocks (blk0-blk15) are highlighted in the LBs. In each cycle, one BRAM address is accessed at most to ensure that the previous constraint of BRAM port is not violated. For the blocks that are not aligned to the BRAM boundary, such as blk3-blk5, a byte-wise write enable signal was used to make sure only the positions marked by the enable signal were updated and the other pixels in the same address are not overwritten. Note that the remainder pixels in the last block of each line are duplicated and stored at the beginning of the successive LB. For example, blk2 contains 4 pixels of Line1 ($N_{remain}=4$). Thus, these pixels are written to both BRAM2 of LB0 and BRAM0 of LB1, concurrently.

Note that from the Blk11, the storage pattern in LBs will be the same as that from Blk0. This is because the values of $N_{remain}$ in continuous lines show a periodic pattern, and the period is determined by $N_{line}$ and $N_{blk}$. The period measured in clock cycle ($P_{clk}$) or in image line ($P_{line}$) is given by Equation (3).

(3) $$\begin{array}{c} P_{clk}=\mathrm{LCM}\left(N_{line},N_{blk}\right)/N_{blk}\\ P_{line}=\mathrm{LCM}\left(N_{line},N_{blk}\right)/N_{line} \end{array}$$

where LCM is the least common multiple. In addition, $N_{remain}$ of line *l* (denoted as $N_{remain,l}$) is calculated as Equation (4).

(4) $$N_{remain,l}=\left\{\begin{array}{cc} N_{blk}-N_{line}\%N_{blk}, & l=0\\ N_{blk}-\left(N_{line}-N_{remain,l-1}\right)\%N_{blk}, & l\in [1,P_{line}) \end{array}\right.$$

where *l* is the index of the line. With the equations above, the buffer access pattern is deterministic. Thus, in the Figure 7 example, every 4 lines have the same LB storage pattern, and the value of $N_{remain,l}$ shows a periodic pattern of {4,8,12,0}.

#### 3.3.2. Control Code Generation

To perform the buffer store and load with the proposed access pattern, D-SWIM adopts customized instructions along with hardware logic to control the LBs. The BRAM control signals inside each LB are given by the instruction codes and decode logic, and the line-rolling behavior (store block/load block) is controlled by the hardware logic. The instruction codes for a specific image size were generated and loaded into the InstructionMemory before run-time. The instruction-based control method has two key benefits: firstly, it saves hardware logic for control signal generation; and secondly, the content in the InstructionMemory can be rapidly switched for processing differently sized images. Note that each instruction manages the buffer behavior over multiple cycles corresponding to one image line.

As Table 2 listed, the customized instruction is composed of five sections, and each of them is translated into specific BRAM control signals by the control logic. Because an arbitrary number of remainder pixels ($N_{remain}$) may exist ahead of the first block of a line (line-initial block), we set section ${\mathrm{MEM}}_{\mathrm{start}}$ to give the BRAM index from which to store the line-initial block. Furthermore, since the block access may not be aligned to the BRAM boundary, the offset position inside a BRAM is given by section ${\mathrm{MEM}}_{\mathrm{offset}}$. In the D-SWIM design, we constrained $N_{blk}$ to be an integer multiple of the BRAM width ($N_{blk}/\left(W_{bram}/8\right)\in Z$). Thus, all pixel blocks in one image line have the same offset inside a BRAM, which is given by ${\mathrm{MEM}}_{\mathrm{offset}}$. This constraint leads to a regular storage pattern and reduces the hardware logic usage for control signal generation. Section REMAIN gives the value of $N_{remain}$, which represents the number of pixels in the last block of a line that overflows into the successive line, and they are duplicated and stored in the next LB. Section CYCLE gives the number of blocks in the line, which indicates the cycle number of the control period for the current instruction code. In addition, CYCLE determines the interval period of fetching a new instruction.

The periodic access pattern of continuous image lines presented in Equation (3) enables instruction reuse. For instance, $P_{line}$ is 4 in Figure 7a; thus, only four instructions are needed. To reuse the instruction periodically, section RETURN gives the flag to reset the instruction-fetch address and restart a new period of the access pattern. The periodic reuse of control code saves the instruction memory and reduces time delay caused by instruction reloading while switching the image size. Theoretically, the maximum possible number of instruction is $N_{blk}$.

Algorithm 1 gives the instruction generation flow in D-SWIM’s SW-tool. Each iteration of the program calculates five sections and then assembles the instruction to binary codes. The iteration continues till $N_{remain}$(value of REMAIN section) gets to zero. In the last instruction of the list, RETURN is set to 1, that leads to re-execution of the entire instruction list. For instance, Figure 7b gives the instruction code for each image line in Figure 7a, which is generated by Algorithm 1. For Line0, the line-initial block starts from BRAM0 without pixel offset. Thus, ${\mathrm{MEM}}_{\mathrm{start}}$ and ${\mathrm{MEM}}_{\mathrm{offset}}$ are 0. It takes 3 blocks to fulfill the Line0, and the last block contains 4 pixels belonging to the following Line1. Thus REMAIN and CYCLE is equal to 4 and 3, respectively. Algorithm 1 starts with an initial state that all variables are set to 0, and input parameters $N_{line}$, $N_{blk}$, and $W_{bram}$ are set to 44, 16, and 64, respectively. In the loop iteration, the variables are calculated sequentially, and they are corresponding to the value of each instruction section for Line0. Then, the values of five sections are assembled into the Instruction0 and appended to the instruction list. Following the Line0, the line-initial block of Line1 starts from BRAM0 of LB1 with a inner-BRAM offset of 4 pixels, which can be translated to ${\mathrm{MEM}}_{\mathrm{start}}$=0 and ${\mathrm{MEM}}_{\mathrm{offset}}$=4 in Instruction1, respectively. The other sections are conducted using the same manner as Instruction0. In particular, only 2 blocks are required to fulfill Line3 (CYCLE in Instruction3=2), because there are 12 remainder pixels contained in the last block of Line2. The Line3 does not contain remainder pixels, that results in pixel blocks from Blk11 which perform the same storage pattern with that of blocks from Blk0. Therefore, in the algorithm loop iteration for Instruction3, variable RETURN is set to 1, and the loop stops. Then, the algorithm outputs the instruction list (Instruction0-3) that can be periodically executed in processing continuous image lines.

**Algorithm 1:** Instruction Generation Algorithm in D-SWIM Streaming Architecture**Input**: Application parameters: $N_{line},N_{blk}$, Hardware information: $W_{bram}$ **Output**: Instruction code: Inst Inst=∅; ${\mathrm{MEM}}_{\mathrm{start}}$ = 0; ${\mathrm{MEM}}_{\mathrm{offset}}$ = 0; REMAIN = 0; RETURN = 0;

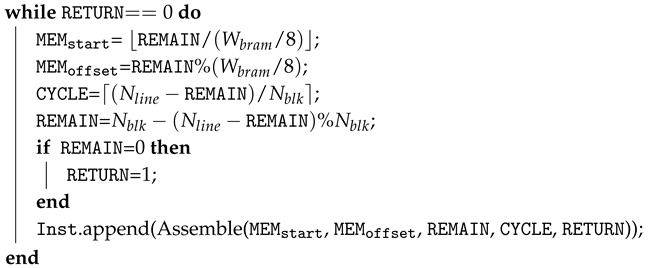
**return**
Inst

### 3.4. Run-Time Dynamic Programming for Arbitrary-Sized Image

**Figure 8:**
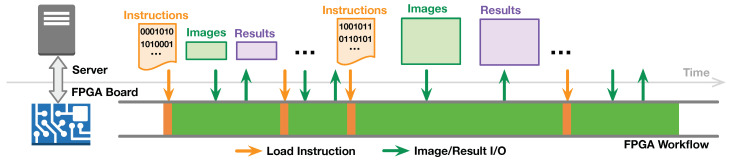
D-SWIM workflow with dynamic programming for arbitrary sized image processing.

With the specific instruction set, the D-SWIM buffer can be rapidly re-programmed for processing arbitrary sized images. Figure 8 demonstrates the system workflow on both the FPGA and the server. The server obtains the images from users and prepares the D-SWIM instruction list for the image size. Due to the low complexity of the instruction generator in Algorithm 1, the server generates the instruction list online and then writes it to the InstructionMemory of D-SWIM at the FPGA side. Besides the instruction, the server also sends the value of image height ($N_{height}$) to the control register in D-SWIM that determines the life-cycle of the instruction list. Subsequently, the server sends the corresponding image to the FPGA and obtains the computational results in a continuous data stream. Note that the communication latency is hidden in the fully pipelined workflow. Thus, the image computation logic on FPGA only stalls for a brief period of time during instruction loading.

## 4. Logic Implementation of D-SWIM

This section explains the detailed implementation of the underlying logic hardware of D-SWIM. Note that the main focus here is a universal buffer design for image-processing-based streaming architectures on modern FPGA architectures. Further optimizations may apply to specific applications on FPGA architectures, but it is outside the scope of this section.

### 4.1. Logic of Line Buffer

**Figure 9:**
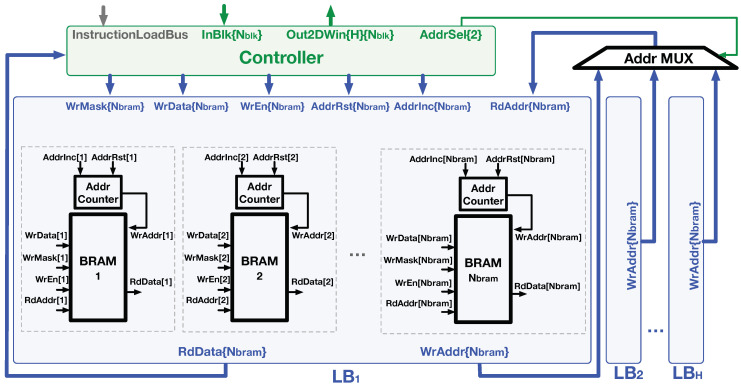
The D-SWIM buffer is composed of LBs and Controller. Each BRAM in the LB is equipped with an AddressCounter to manage the write address. It performs address incrementation or reset according to the signal on the controller bus. The AddrMUX allows the write addresses to be broadcasted during a block write operation of a specific LB as the read addresses of the other LBs for block loading.

Figure 9 shows the hardware composition of the Line Buffer (LB) and the related control signals from the Controller module. Each LB is composed of $N_{bram}$ BRAMs and the associated control logic. According to the LB access pattern described in Section 3.3, the BRAM addressing pattern is sequential. Thus, we employed an AddrCounter module to each BRAM to manage the write address. The AddrCounter accepts the AddrInc and AddrRst signals from the Controller that determines whether to increase the address register by one or reset it to zero, respectively. The other signals related to the block store process, including WrMask, WrData, and WrEn, were generated by the Controller and directly connected to the BRAM primitives. Note that we annotate the width of each signal bus in the brace following the signal name in Figure 9.

According to the line-rolling buffer access behavior, the read addresses of multiple LBs were synchronized to the write address of the LB which stores the input block. In the logic design, the WrAddr from AddrCounters were sent to a MUX, and the MUX selected the proper value as the RdAddr signal of all LBs under the control of the AddrSel signal.

### 4.2. Logic of Controller

The Controller performs three functions in D-SWIM: (1) decode the instruction word to the control signals of each LB; (2) transform the input pixel block (InBlk) to the proper storage pattern as the WrData signal of the BRAMs; (3) transform the pixels loaded from LBs to the certain 2D window required by the operators (Out2DWin). Thus, the buffer-write and buffer-read logic are implemented independently as follows.

#### 4.2.1. Buffer-Write Logic

**Figure 10:**
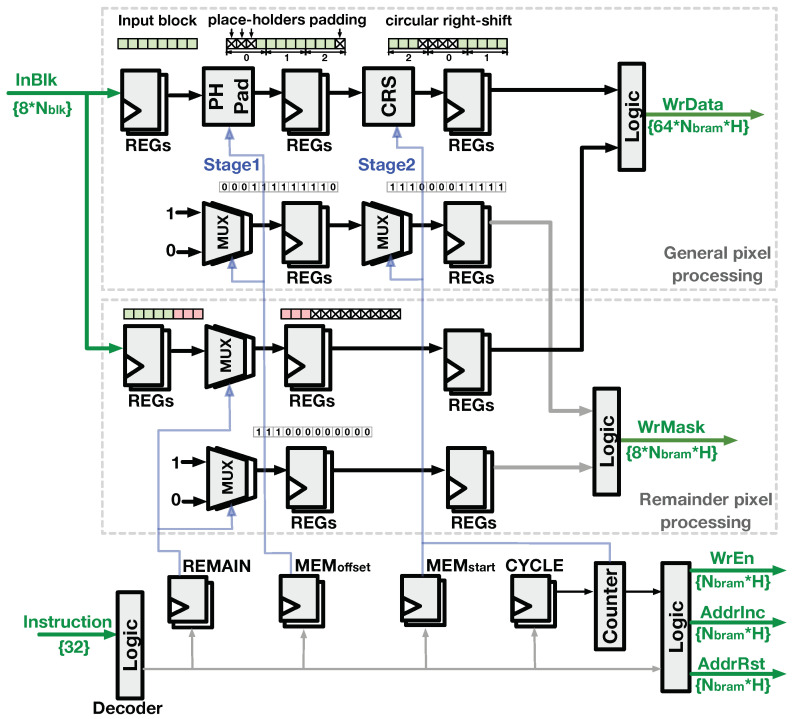
Buffer-write logic.

In the D-SWIM design, the length of input block (InBlk) is larger than the width of LB. Thus, the buffer-write logic extends the InBlk signal to the same width of LB in a certain pattern, and the BRAM byte-wise write enable signal (WrMask) is generated concurrently. As Figure 10 shows, two stages exist in the signal generation. In the first stage, place-holding bytes (cross marked in Figure 10) are padded at the beginning and the end of the input block. By doing so, the padded block has the same width as the LB. The number of place-holders at the start of the block is equal to ${\mathrm{MEM}}_{\mathrm{offset}}$ in the instruction word. Thus, the PHPad hardware in this stage is controlled by the corresponding register decoded from the instruction. In the second stage, the pixels from the first stage are rearranged by a circular right-shift (CRS) operator. The shift distance is an integer multiple of the BRAM width, and it is given by ${\mathrm{MEM}}_{\mathrm{start}}$ in the instruction that ensures the pixel-block storage starts from the proper BRAM in the LB. Note that the pattern of place-holder padding is fixed for blocks in the entire image line, but the shift distance in the second stage changes for every block. Thus, the CRS hardware in the second stage is controlled by the ${\mathrm{MEM}}_{\mathrm{start}}$ and a run-time Counter which provides the input block index of one image line. Along with the WrData signal, the WrMask signal is generated in a similar manner. After the two-stage processing, a set of binary flags are generated, where 0 corresponds to the positions of the place-holders in WrData and 1 indicates that the BRAM byte position will be overwritten by the value in WrData.

In particular, when the input block exceeds the end of the image line, the logic stores the *remainder pixels* belonging to the next image line to the beginning of the next LB concurrently, where specific logics are set to process these remainder pixels. Because the number of remainder pixels is provided by the REMAIN section in the instruction, the MUX in the logic separates remain pixels (red pixels in Figure 10) and pads place-holders in the tail as the WrData signal of the next LB, while the WrMask signal with $N_{remain}$ ones at the beginning of the binary set is generated. Subsequently, the WrData and WrMask signals from the circuits for general pixels and remainder pixels are concatenated as the output bus of the Controller. Other buffer-write related signals, WrEn, AddrInc, and AddrRst, were generated concurrently by the Counter and specific logics.

#### 4.2.2. Buffer-Read Logic

**Figure 11:**
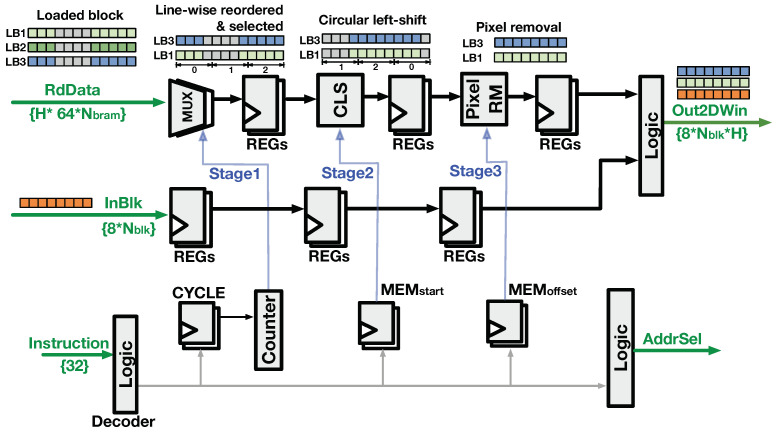
Buffer-read logic.

As introduced previously, the BRAM read address was synchronized to the write address of the LB being written. Thus, the Controller generates the AddrSel signal to indicate the LB index that stores the input block. The RdData loaded from multiple LBs are processed by the buffer-read logic to form the output 2D pixel window (Out2DWin) from multiple image lines. The buffer-read logic reverses transformation performed during the buffer-write process, and the circuit is shown in Figure 11. The logic contains three stages to transform the RdData into Out2DWin. The first stage performs line-wise reordering that changes the line-order of the LBs’ output blocks to the spatial order of the image. As per the line-rolling behavior in Figure 6, (H-1) blocks of Out2DWin are read from the LBs, and the last block is directly sourced from InBlk with delay logic. The second stage performs a circular left-shift (CLS) which reorders the pixels from different BRAMs. The third stage removes several pixels at the beginning and the end of results from the previous stage, which ensures the output blocks are spatially aligned to the InBlk. Subsequently, the pixel blocks after the three-stage processing are concatenated with the delayed InBlk to construct the Out2DWin.

## 5. Evaluation

This section describes the experimental setup for evaluating the D-SWIM implementation. In the evaluation, we compare this work with SWIM in terms of hardware resource usage, timing, and power consumption on FPGA. Subsequently, we evaluate the D-SWIM workflow with dynamic programming for continuously processing images in different sizes. Furthermore, we present D-SWIM based hardware architectures for two real-world image applications (2D-Convolution and Harris Corner Detector). The implementations are compared to streaming architectures in prior works.

### 5.1. Experiment Setup and Evaluation Metric

The hardware generator of D-SWIM was realized using Verilog RTL and implemented with Xilinx Vivado. Our selected target FPGA device was Xilinx-XC7VX690, where the tool synthesizes the Verilog into logic circuits and generates FPGA-specific mapping. Meanwhile, it also gives the resource utilization and timing performance, which are generally employed as the evaluation metrics of FPGA implementation. The resource utilization can be broken down into four FPGA building-blocks: the look-up table (LUT), Flip-Flop Register (REG), BRAM, and DSP. On the timing performance, the worst negative slack (WNS) of the critical path is given by Vivado and can be translated to the maximum operating frequency ($f_{max}$). The vendor tool gives the power consumption of the D-SWIM module as well. Besides the hardware tools, we implemented the D-SWIM instruction generator on the server to provide the control codes for any given image width ($N_{line}$) and block size ($N_{blk}$).

### 5.2. Evaluation of Buffer Hardware

We evaluated the buffer design in D-SWIM and compared it with the previous work of SWIM, which tackles the similar BRAM-misalignment issue for the multi-pixel block but only supports static image sizes that are pre-defined before FPGA synthesis. In the experiment, we configure the SWIM and D-SWIM with different parameter sets for a complete evaluation. As Table 3 shows, the parameters ($N_{line}$, *H*, and $N_{blk}$) are set to different values, and the implementation results are listed. In Configurations 1–6, we set the image width ($N_{line}$) to arbitrary values. The window height (*H*) was set to 3 or 5, which are frequently used in image applications. The pixel number of one input block ($N_{blk}$) was set to 8,16,32. Note that in the SWIM method, the number of LB ($N_{LB}$) was deduced by $N_{blk}$ and $N_{line}$; thus, it may exceed *H*, whereas $N_{LB}$ is equal to *H* in D-SWIM. The optimization technique of multi-pass BRAM partitioning in SWIM was also invoked to reduced $N_{LB}$, and the corresponding results are shown in the SWIM-2pass column of Table 3.

#### 5.2.1. Resource Evaluation

Table 3 lists the resource consumption of LUT, REG, and BRAM for different buffer schemes. Note that the results of D-SWIM and SWIM-2pass schemes are compared, and the ratio values of D-SWIM to SWIM-2pass are listed in the parenthesis of the D-SWIM column. In Configuration 1, D-SWIM consumes 44.9% and 34.7% more LUT and REG than that in SWIM. However, the BRAM consumption in D-SWIM is less—this is because $N_{LB}$ is 4 in SWIM as 4 lines compose a BRAM-partition period, but this issue does not exist in D-SWIM. When $N_{blk}$ is set to 16, as per Configuration 2, $N_{LB}$ of SWIM increases to 8, which consumes more logic and BRAMs than D-SWIM. Although the 2-pass optimization halves the $N_{LB}$, the BRAM cost in SWIM is 16, which is 1.8 times that of 9 in D-SWIM. SWIM costs more BRAMs of specific widths to compose the LB, whereas D-SWIM sets all BRAM ports to the maximum width, which fully utilizes the bandwidth and reduces the resource. Note that the D-SWIM buffer accommodates arbitrary image width, and configurations use different $N_{line}$ values but identical *H* and $N_{blk}$ (e.g., Configuration 2 and Configuration 3) share the same hardware via dynamic programming. In Configurations 5–6 where *H* is 5, D-SWIM’s consumptions of both logic and BRAM are less than SWIM. D-SWIM saves 13.1% LUT, 31.4% REG, and 55.9% BRAM compared with SWIM-2pass in Configuration 6. With Configuration 6, the $N_{LB}$ of SWIM is 8, which costs more logic on the line-selection multiplexer.

We also investigate the impact of parameters on the resource consumption in the D-SWIM scheme. Comparing the results in Configurations 1–4, we note that $N_{blk}$ greatly affects the logic and BRAM resources. This is because a larger $N_{blk}$ requires more complex multiplexers for pixel-block manipulation. Note that *H* also affects the hardware (comparing Configuration 3 to Configuration 5) because a larger *H* costs more LUTs for the line-selecting multiplexer and more REGs on the temporary pixel-storage.

#### 5.2.2. Timing and Power Evaluation

**Figure 12:**
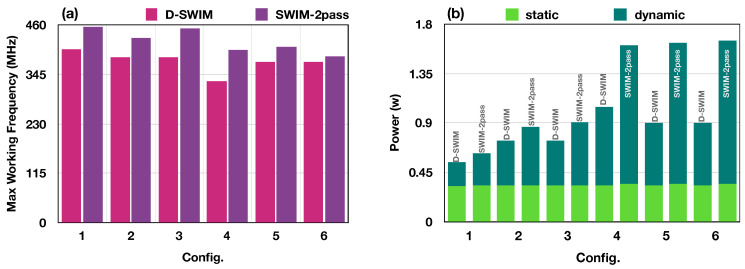
(**a**) shows the $f_{max}$ of D-SWIM and SWIM designs with the configurations in Table 3. (**b**) shows the power consumption of D-SWIM and SWIM, with the breakdown of static and dynamic power.

In addition to the hardware resource usage, timing performance and power consumption were also evaluated. The results of the post place-and-route design were obtained from the vendor tools (Xilinx Vivado) and presented in Figure 12, where (a) is the $f_{max}$ with different configurations in Table 3 and (b) is the power consumption. Compared with SWIM, the D-SWIM design slightly decreases the $f_{max}$. This is because the multi-stage logics for dynamic controlling lengthen the critical path. We selected the proper pipeline stage as described in Section 4, while trading the $f_{max}$ and resource overhead in further pipelining. We observed that $N_{blk}$ is a significant factor to the $f_{max}$. In Configurations 1–6, the worst $f_{max}$ is 329.5 MHz with Configuration 4 in which $N_{blk}$ is 32.

Figure 12b presents the power consumption of buffer modules with SWIM and D-SWIM design methods. Each power bar is composed of two portions that represent static power (lighter part) and dynamic power (darker part). Apparently, SWIM and D-SWIM have identical static power with all configurations, but D-SWIM performs better in the dynamic power aspect. This is mainly due to fewer LBs ($N_{LB}$) used in the D-SWIM case, which leads to less BRAM usage. For example, with Configuration 5, SWIM consumes 2.1× BRAMs of that in D-SWIM; thus, the dynamic power is increased proportionally. Compared with SWIM, the D-SWIM buffer saves up to 45.7% power consumption in the case of Configuration 6.

### 5.3. Evaluation of Dynamic Programming in D-SWIM

The dynamic programming described in Section 3.4 contributes to the ability of rapid context-switching for arbitrary sized images. We evaluated the D-SWIM system with the workload containing images in different sizes. Table 4 lists the period of computation and dynamic programming for one image. The independent variables are *image size* and $N_{blk}$ (input throughput), which affects the measured periods. The values were measured in clock cycles. However, for direct comparison, they were converted to the numbers in micro-seconds with an operating frequency of 350 MHz. The *proportion* column gives the ratio of the programming period to the entire working period (programming period + computation period). The overhead of dynamic programming in D-SWIM is significantly less than 1% in most cases, and the context-switching can be regarded as seamless.

In contrast, while employing the SWIM buffer for the same workload, a FPGA reconfiguration is required to switch the specific hardware corresponding to the size of the input image. The average period for reconfiguring the entire FPGA device (Xilinx-XC7VX690) is 20 s. For a fair comparison, we listed the time period for the partial reconfiguration technique [14], which reduces the reconfiguration time to the order of milliseconds via programming only a portion of the FPGA. Similarly, we obtained the proportion of FPGA reconfiguration time based on the image-processing period. Assuming the SWIM hardware is reconfigured for each image, the results show that the FPGA performs reconfiguration in over 80% of the entire working period. This means that the FPGA spends the most time on reconfiguration rather than actual image processing, causing a huge reduction in processing throughput.

### 5.4. Case Study of Image Processing with D-SWIM

With the D-SWIM buffer, an architecture for a specific image application can be easily constructed. This section presents D-SWIM-based architectures for two real-world image applications and their performance study.

To evaluate the practicability of D-SWIM in real applications, we compare the D-SWIM designs with similar streaming architectures for image processing [7,10]. In prior studies, Reiche et al. [7] improved the ${\mathrm{HIPA}}^{\mathrm{CC}}$ image-processing framework to generate effective High-level Synthesis (HLS) codes for FPGA with a specific memory architecture, and Özkan et al. [10] optimized the OpenCL framework of Intel (Altera) FPGA to a domain-specific language (DSL) for image processing. These works are widely accepted by the community, and were developed based on the latest tools from the industry and academia. Thus, we consider these two as the state-of-the-art works for comparison.

#### 5.4.1. Conv2D

**Figure 13:**
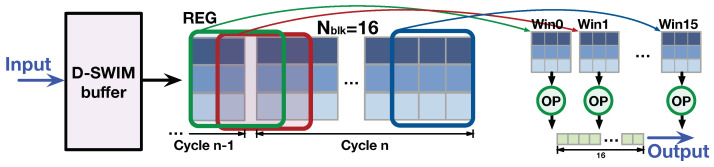
D-SWIM-based architecture for Conv2D (3×3 window).

2D convolution (Conv2D) is a popular operation for image feature extraction. Figure 1 shows a typical Conv2D operation with 3×3 convolutional kernels. Pixels in a kernel-sized window were fetched and multiplied to the kernel weights and then accumulated to the Conv2D result. The subsequent operation moves the window 1 pixel to the right, and performs the same arithmetic. In the high-throughput scenario of D-SWIM, multiple overlapped windows are processed in the same clock cycle. Thus, the logic components are connected as Figure 13. In every clock cycle, the buffer output pixel block has a height of *H*, and width of $N_{blk}$. The pixels were directly delivered to the parallel operators (OP) which performed the MACC operation. The results of OPs were concatenated to the output block. Note that there are windows, such as Win0 and Win1, in Figure 13, containing pixels from two consecutive output blocks of the buffer. Thus, we set registers to store the last pixel-columns of the previous cycle to construct these windows.

Following the architecture above, we implemented the Conv2D with $N_{blk}=16$ and H=3. For a fair comparison, the hardware of OP was simply implemented with naïve RTL with a pre-defined pipeline stage. The implementation results are listed in Table 5. We name the works in [7,10] Design2 and Design1, respectively, in the following content. The devices adopted in each work have been listed as a reference. Note that the size of BRAM in Intel FPGA is 20 Kbits, whereas it is 36 Kbits in Xilinx FPGA. $N_{blk}$ represents the hardware throughput (pixel/cycle). Meanwhile, the $f_{max}$ of each design has been given, and we obtained the system pixel throughput (giga pixel per second (GPPS)) by $N_{blk}\times f_{max}$.

Because D-SWIM and Design1–2 have different throughputs, it was unfair to compare the resource number in Table 5 directly. Thus, we obtained the hardware efficiency of FPGA logic (LUT and REG) with Equation (5). Comparing with the highest-throughput design (Design1), the hardware efficiency of D-SWIM is 4.8× and 8.2× in LUT and REG, respectively. Comparing with the smallest design (Design2), D-SWIM also achieves competitive hardware efficiency. Note that Design2 achieves higher hardware efficiency on LUT, as it has traded off throughput severely for simpler logic. Moreover, it does not consider the issues in the multi-pixel input scenario (such as BRAM-misalignment), which allows further reduction in overall hardware usage.

(5) $$Efficiency=Throughput/Hardware\;Consumption\times 10^{5}$$

#### 5.4.2. Harris Corner (HC) Detector

The Harris Corner (HC) detector [15] is commonly used in computer vision applications that detects the corner position for feature matching. The HC operator swaps the window (e.g., 3×3 pixels in the benchmark) on the image and determines if the window contains a corner pattern. The HC algorithm on each window is listed in Equation (6). Firstly, HC obtains the gradient matrix M, where I(i,j) is the intensity value of the pixel inside the window; $\frac{\partial I(i,j)}{\partial x}$ and $\frac{\partial I(i,j)}{\partial y}$ are the intensity derivative in the horizontal and vertical axes, respectively. Secondly, *R* was calculated to estimate the eigenvalue of M, where *k* is a constant of 0.04–0.06, and det and trace calculates the determinant and trace of the matrix, respectively. If the value of *R* is larger than the threshold, the current window contains the corner pattern.

(6) $$\begin{array}{cc} M= & \left[\begin{array}{cc} \sum_{i,j}^{W}{\left(\frac{\partial I(i,j)}{\partial x}\right)}^{2} & \sum_{i,j}^{W}\left(\frac{\partial I(i,j)}{\partial x}\right)\left(\frac{\partial I(i,j)}{\partial y}\right)\\ \sum_{i,j}^{W}\left(\frac{\partial I(i,j)}{\partial x}\right)\left(\frac{\partial I(i,j)}{\partial y}\right) & \sum_{i,j}^{W}{\left(\frac{\partial I(i,j)}{\partial y}\right)}^{2} \end{array}\right]\\ R= & \det\left(M\right)-k\times \mathrm{trace}{\left(M\right)}^{2} \end{array}$$

**Figure 14:**
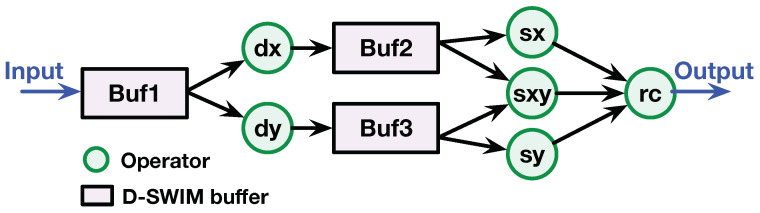
D-SWIM-based architecture for HC detector (3×3 window).

Figure 14 demonstrates the D-SWIM-based architecture for HC. This streaming architecture is composed of a buffer (rectangular shape), operator (circular shape), and interconnections. Note that the derivative calculation in different axes can be realized by a Conv2D operation with the Sobel kernels. We used a 3×3 Sobel kernel in the example, and the operators are denoted as dx and dy for the two axes. The derivative results ($\frac{\partial I(i,j)}{\partial x}$, $\frac{\partial I(i,j)}{\partial y}$) were stored in Buf2 and Buf3 because they were accessed with a 2D window pattern in the subsequent operations. The sx, sy, and sxy operators perform the element-wise multiplication and accumulate the values in the window. After M is obtained, operator rc calculates the *R* in Equation (6) and compares it with the threshold to determine whether the window contains a corner or not.

The evaluation method of HC is the same as the Conv2D case. Table 6 shows the implementation results of D-SWIM and Design1–2. In the HC case, the D-SWIM-based design achieves both the highest throughput and the best hardware efficiency. Comparing with the superior design (Design1), D-SWIM increases the throughput to 3.5×. Furthermore, with D-SWIM, the efficiency of LUT and REG is 25× and 30× that in prior studies.

## 6. Conclusions

This work has presented D-SWIM, a dynamic programmable line buffer microarchitecture for arbitrary sized streaming image processing on FPGAs. The D-SWIM architecture facilitates high-throughput realignment of multi-pixel blocks into line buffers suitable for further streaming image processing. In addition, through a rapid instruction code update, D-SWIM allows for the size of line buffers to adjust dynamically to accommodate varying size requirements of the application during run time. Compared to prior studies where SWIM can only work on a predetermined image size, D-SWIM achieves dynamic programmability for varying image sizes with a slight logic resource overhead. In our experiment, the D-SWIM buffer reached a maximum operating frequency of 329.5 MHz and saved BRAM resources up to 56% that contributed to a power consumption reduction of 45.7%. When compared to other state-of-the-art FPGA-based streaming architectures using two real-world image applications as benchmarks, D-SWIM contributes to a significant hardware efficiency improvement of 25× in LUT and 30× in REG. For the benchmark cases, the D-SWIM based design reaches a pixel throughput of 4.2 GPPS when 16 pixels are input every cycle.

As more applications domain begin to take advantage of vision-based intelligence, the number of systems that demand high-performance image processing is going to increase. D-SWIM represents our first step in systematically generating flexible high-throughput low-latency streaming image processing hardware. In the future, we expect to further the capability of D-SWIM to facilitate generation of the complete intelligent image processing system automatically for FPGAs.

## Figures and Tables

**Table 1:** Design parameters in D-SWIM framework.

Design Parameters	Description	Use Scope
$N_{blk}$	Number of pixels in one stream block	HW & SW
$N_{line}$	Number of pixels in the image line (image width)	SW
$N_{height}$	Image height	SW
$N_{line-max}$	Largest possible value of $N_{line}$	HW
*H*	Height in the vertical axis of the 2D stencil pattern	HW

**Table 2:** Sections of the customized instruction for D-SWIM architecture.

Section	Bit-Length	Description
${\mathrm{MEM}}_{\mathrm{start}}$	$\left\lceil \log_{2}N_{bram}\right\rceil$	Start BRAM index of line-initial block
${\mathrm{MEM}}_{\mathrm{offset}}$	$\left\lceil \log_{2}W_{bram}\right\rceil$	Start position (inside BRAM) of line-initial block
REMAIN	$\left\lceil \log_{2}N_{blk}\right\rceil$	$N_{remain}$ of the current image line
CYCLE	$\left\lceil \log_{2}\left\lceil N_{line-max}/N_{blk}\right\rceil \right\rceil$	Number of blocks in the current image line
RETURN	1	Flag to reset the instruction-fetch address

**Table 3:** Buffer resource consumption in SWIM and D-SWIM.

Config.	Parameters			D-SWIM				SWIM				SWIM-2pass			
	$N_{\mathrm{line}}$	H	$N_{\mathrm{blk}}$	$N_{\mathrm{LB}}$	LUT	REG	BRAM	$N_{\mathrm{LB}}$	LUT	REG	BRAM	$N_{\mathrm{LB}}$	LUT	REG	BRAM
1	630	3	8	3	1950 (1.45)	2140 (1.35)	6 (0.75)	4	1346	1589	8	4	1346	1589	8
2	630	3	16	3	3427 (1.33)	3553 (1.29)	9 (0.56)	8	3895	5127	32	4	2650	2653	16
3	1020	3	16	3	3427 (1.30)	3553 (1.16)	9 (0.56)	4	2643	3051	16	4	2643	3051	16
4	1020	3	32	3	7656 (1.18)	6338 (0.94)	15 (0.47)	8	8745	12662	60.5	4	6491	6743	32
5	1020	5	16	5	5608 (1.04)	4931 (0.80)	15 (0.47)	8	5367	6142	32	8	5367	6142	32
6	1375	5	16	5	5608 (0.87)	4931 (0.69)	15 (0.44)	16	10,833	13,305	64	8	6452	7182	34

**Table 4:** Time period of dynamic programming of D-SWIM and partial reconfiguration of SWIM.

Image Size	$N_{\mathrm{blk}}$	Computation Time		D-SWIM Programming Time			SWIM Reconfiguration Time		
H×W (pixel)	(pixel)	(cycle)	(μs)	(cycle)	(μs)	Proportion	(cycle)	(μs)	Proportion
431 × 392	8	21,227	60.649	8	0.023	0.04%	465,500	1330	95.64%
431 × 392	16	10,614	30.326	16	0.046	0.15%	465,500	1330	97.77%
431 × 392	32	5307	15.163	32	0.091	0.60%	465,500	1330	98.87%
1342 × 638	8	107,360	306.743	4	0.011	0.00%	465,500	1330	81.26%
1342 × 638	16	53,680	153.371	8	0.023	0.01%	465,500	1330	89.66%
1342 × 638	32	26,840	76.686	16	0.046	0.06%	465,500	1330	94.55%

**Table 5:** Hardware resource consumption and throughput in a Conv2D implementation.

Work	Device	$N_{\mathrm{blk}}$	Precision	Hardware Consumption				$f_{\max}$	Throughput	Efficiency	
		(pixel)	(bit/pixel)	LUT	REG	BRAM	DSP	(MHz)	(GPPS)	LUT	REG
Design1 [10]	Intel-5SGXEA7	32	8	47,045	73,584	363	0	303.6	9.71	20.7	1.3
Design2 [7]	Xilinx-XC7Z045	1	8	288	521	2	0	349.9	0.35	121.5	6.7
D-SWIM	Xilinx-XC7VX690	16	8	4514	4232	9	76	283	4.5	100.3	10.7

**Table 6:** Hardware resource consumption and throughput in HC detector implementation.

Work	Device	$N_{\mathrm{blk}}$	Precision	Hardware Consumption				$f_{\max}$	Throughput	Efficiency	
		(pixel)	(bit/pixel)	LUT	REG	BRAM	DSP	(MHz)	(GPPS)	LUT	REG
Design1 [10]	Intel-5SGXEA7	4	8	135,808	192,397	493	36	303.4	1.2	0.9	0.1
Design2 [7]	Xilinx-XC7Z045	1	8	23,331	31,102	8	254	239.4	0.24	1.0	0.1
D-SWIM	Xilinx-XC7VX690	16	8	16769	14439	27	444	267	4.2	25.5	3.0

## Abbreviations

The following abbreviations are used in this manuscript:

BRAM	Block Random Access Memory
CLS	Circular Left Shift
CRS	Circular Right Shift
DFG	Data Flow Graph
DNN	Deep Neural Networks
DRAM	Dynamic Random Access Memory
DSL	Domain Specific Languages
DSP	Digital Signal Processing
FIFO	First-In, First-Out
FPGA	Field Programmable Gate Array
GPPS	Giga Pixels Per Second
GOPS	Giga Operations Per Second
LB	Line Buffer
LUT	Look-up Table
REG	Register
ROI	Region of Interest
RTL	Register Transfer Level
SDP	Simple Dual Port
SWIM	Stream-Windowing Interleaved Memory
TDP	True Dual Port
